# Potential Prognostic Parameters from Patient Medical Files for Inhalation Injury Presence and/or Degree: A Single-Center Study

**DOI:** 10.3390/ebj7010002

**Published:** 2025-12-22

**Authors:** Tarryn Kay Prinsloo, Wayne George Kleintjes, Kareemah Najaar

**Affiliations:** 1 Department of Biomedical Sciences, Faculty of Health and Wellness Sciences, Cape Peninsula University of Technology (CPUT), Cape Town 7535, South Africa; tarrynkayprinsloo@gmail.com; 2 Department of Surgical Sciences, Faculty of Medicine and Health Sciences, Stellenbosch University (SU) Medical School, Francie van Zijl Avenue, Cape Town 7505, South Africa; waynekleintjes@yahoo.com; 3 Western Cape Provincial Adult Tertiary Burns Centre, Tygerberg Hospital, Tygerberg, Francie van Zijl Avenue, Cape Town 7505, South Africa; 4 Department of Wellness Sciences, Faculty of Health and Wellness Sciences, Cape Peninsula University of Technology (CPUT), District Six 8000, P.O. Box 652, Cape Town 7530, South Africa

**Keywords:** inhalation injury, prognosis, association, correlation, regression, burns, Western Cape Provincial Adult Tertiary Burn Centre, Tygerberg Hospital

## Abstract

(1) Background: Inhalation injury significantly worsens burn outcomes but lacks a standardized definition and diagnostic consensus, complicating prognosis. Existing diagnostic tools often show limited sensitivity and specificity, reducing clinical utility. This study aimed to identify potential clinical markers, recorded at or shortly after admission, for inhalation injury prognostication. (2) Methods: A retrospective cohort study of 59 burn patients admitted to Tygerberg Hospital’s Burn Centre (South Africa) between 23 April 2016 and 15 August 2017 was conducted. Descriptive statistics were reported based on data type and distribution. Fisher’s exact test, Spearman’s rank correlation (rho), and partial least squares regression (VIP scores) assessed associations, correlations, and predictive value. *p* < 0.05 (two-tailed) denoted significance. (3) Results: Severe inhalation injury accounted for 61% of admissions (mean 11.2; CI = 9.5–12.9), with a 38.9% mortality rate. Significant associations (*p* ≤ 0.008) and positive correlations (*p* ≤ 0.06) were noted for total body surface area (rho = 0.357), complications (rho = 0.690), and burns intensive care unit length of stay (BICU LOS, rho = 0.908). Complications and BICU LOS showed the strongest predictive contributions (VIP = 1.229 and 1.372). Lactate (rho = 0.331, *p* < 0.011) and hoarseness (rho = −0.314, *p* < 0.015) correlated significantly but lacked association. (4) Conclusions: Findings suggest elevated lactate may serve as a prognostic marker, while BICU LOS and complications may reflect disease progression. A multi-marker approach is recommended.

## 1. Introduction

Inhalation injury refers to respiratory tract damage following exposure to toxic smoke inhalants during thermal events, such as domestic fires, self-immolation, or steam accidents [1,2]. Resultant injuries range from supraglottic irritation to severe systemic toxicity induced by chemical exposure [3,4,5], with the latter representing the most critical clinical presentations. Despite advancements in supportive care and critical care interventions, morbidity and mortality rates associated with inhalation injury remain high [6,7]. Thus, it has been recognized as one of the most significant cofactors in burn-related mortality [7,8], independently increasing the risk thereof by 20% [5,8], elevating morbidity [7], and increasing the risk for complications that ultimately impact patient outcomes [9]. The clinical presentation of inhalation injury is highly variable, influenced by factors such as the chemical composition of smoke, fire origin, exposure duration, and patient-specific comorbidities [2,5,10]. These variables contribute to the latent and often non-specific manifestation of symptoms, which can appear within 24–48 h post-exposure [11,12], but may be delayed up to four days [13]. Additionally, complications may present acutely or be delayed [2,14], depending on the severity of the injury, management strategies, and persistent inflammatory responses [2,15]. These complexities complicate both diagnosis and prognosis [4], increasing the likelihood of misdiagnosis [12].

Current diagnostic approaches vary across clinical settings but generally rely on a combination of subjective and objective assessments—including history evaluation, physical examination, and a high index of clinical suspicion [16,17,18]. In the absence of standardized diagnostic criteria, these clinical evaluations serve as the primary assessment tool, with confirmatory diagnostics employed as secondary measures [19]. Although diagnostic evaluations are central to burn care, their ability to reliably predict outcomes remains limited [20]. Moreover, in resource-constrained settings, such as Tygerberg Hospital (TBH, Cape Town, South Africa), the associated financial and logistical constraints further limit access to specialized diagnostic tools required for grading systems such as the Abbreviated Injury Scale [21] and the Respiratory Acute Dysfunction Score [22]. Thus, management and treatment of inhalation injury has largely been supportive [13], and given its reported prevalence in approximately one-third of patients admitted to TBH [23,24,25], the need for accurate prognostic indicators is particularly important. Considering initial mortality risk assessments in burn patients routinely include indicators suggestive of inhalation injury, such as facial burns, hoarseness, and stridor [2,5], we postulated that additional routinely recorded clinical parameters, traditionally considered only in the context of mortality, may also hold prognostic value for inhalation injury. The use of routinely collected clinical data offers a practical advantage due to its immediate availability to clinicians as part of standard admission and assessment procedures. The use of such readily available clinical data offers a practical and cost-effective approach, particularly in settings with limited diagnostic capacity. This study therefore aimed to investigate associations between inhalation injury and routinely recorded clinical parameters, with the objective of identifying potential prognostic markers for its presence and/or severity.

## 2. Materials and Methods

### 2.1. Study Design and Setting

**Figure 1:**
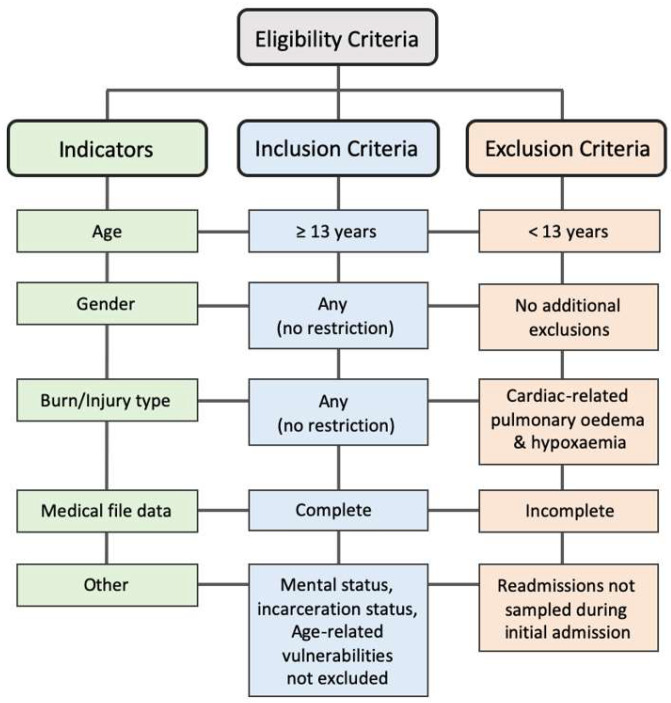
Eligibility criteria of the study comprising the inclusion and exclusion indicators.

This was a retrospective cohort study of burn patients (*n* = 59) admitted to the Western Cape Provincial Adult Tertiary Burns Centre (WCPATBC) at TBH, South Africa, between 23 April 2016 and 15 August 2017. TBH is the larger of two adult tertiary hospitals in the Western Cape (WC) with a dedicated burn referral center [26] and has been reported to have the highest rate of fire-related fatalities in the region [27]. According to the admission criteria, individuals aged 13 years and older are treated as adults at the WCPATBC [28,29], while the Red Cross Children’s Hospital accepts patients younger than 13 years [30]. Only approximately 20% of patients admitted to the WCPATBC fall within the 13–20-year age range (per WCPATBC unit specialist, personal communication, 2025). Following ethical approval and informed consent, patient data were extracted from the internal WCPATBC database by a medical officer using reference numbers to ensure de-identification and anonymity [31]. The inclusion criteria encompassed all adult burn patients (≥13 years) with complete medical files, irrespective of gender, mental status, incarceration status, or age-related vulnerabilities, and regardless of burn type. Exclusion criteria included patients under 13 years of age; readmitted patients who were not sampled during their initial admission; patients presenting with cardiac-related pulmonary edema and hypoxemia; and cases with incomplete medical files, which were excluded to preserve statistical integrity (Figure 1).

### 2.2. Data Collection and Sorting

Demographic information, injury characteristics, and clinical parameters were categorized into sub-groups for analysis. Demographic variables included sex (male or female), age group (13–20, 21–39, 40–49, and ≥50 years), day of the week (weekday vs. weekend), seasonal variation (colder vs. warmer seasons), referral setting by district (City of Cape Town-CoCT vs. other WC districts), and level of referring facility (hospital vs. clinic or community health center). Injury characteristics encompassed burn etiology (fire vs. other), total body surface area burned (%TBSA: ≤40% or >40%), phenotypical indicators suggestive of inhalation injury (the presence of singed nasal hairs, soot in or around the mouth, hoarseness, and facial burns), complications (yes/no), and Abbreviated Burn Severity Index (ABSI) scores, which were grouped as follows [32]: moderate (4–5), moderately severe (6–7), serious (8–9), severe (10–11), and maximum severity (12–13). Complications included ventilator-associated pneumonia (VAP), aspirated pneumonia, septicemia, septic shock, acute respiratory distress syndrome (ARDS), and the combined presence of septic shock and ARDS, as recorded in the medical files (no additional information on the diagnostic criteria applied was available). Clinical parameters included length of stay (LOS) in the Burns Intensive Care Unit (BICU), stratified as 0–9 or ≥10 days; pre-admission ventilation (yes/no); and arterial blood gas (ABG) metrics, including blood pH (<7.35, 7.35–7.45, >7.45), partial pressure of oxygen (PaO_2_: <10.5, 10.5–13.5, >13.5 kPa), partial pressure of carbon dioxide (PCO_2_: <4.7, 4.7–6.0, >6.0 kPa), oxygen saturation (Sats: <95% vs. 95–100%), lactate levels (<2.0 vs. ≥2.0 mmol/L), and base excess (BE: <−4, −4 to +2, >2 mmol/L), with cut-offs selected according to standard clinical reference ranges. The degree of inhalation injury was stratified by total days of ventilation, with 0–4 days indicating mild injury, and ≥5 days indicating severe injury. Mortality rate was calculated as a percentage of the respective sub-group. Continuous variables, including age, %TBSA, ABSI scores, length of stay, days of mechanical ventilation, and ABG parameters, were therefore stratified into clinically meaningful categories or ordinal groups to enable statistical analysis, while categorical and binary variables (e.g., sex, burn etiology, complications, presence/absence of inhalation injury indicators, mortality) were statistically analyzed as such.

### 2.3. Statistical Analysis

All statistical analyses were conducted using IBM SPSS Statistics version 23 (IBM Corp., Armonk, NY, USA). Descriptive statistics were reported as incidence, frequency, and proportions [patients, N (%)]. Additionally, central indices were reported as mean with 95% confidence intervals (95% CI) or median with interquartile range (IQR), depending on data type and distribution. Normality was assessed using the D’Agostino–Pearson and Shapiro–Wilk tests. Subsequent statistical testing was guided by whether assumptions for parametric or non-parametric methods were met or violated. Fisher’s exact test was used to assess associations between inhalation injury and the variables. The Spearman’s rank correlation coefficient (rho) with 95% CI was used to evaluate the strength and direction of correlations. Correlation strength was interpreted as follows: 0.01–0.19 = negligible, 0.20–0.29 = weak, 0.30–0.39 = moderate, 0.40–0.69 = strong, and ≥0.70 = very strong. Direction was based on positive or negative rho values [33]. All statistical significance levels were two-tailed, with *p*-values < 0.05 considered significant. Partial least squares (PLS) regression was applied to identify which significantly correlated independent explanatory variables (X) best predicted the dependent outcome variable (Y: inhalation injury) [34]. The latent components of X that accounted for the greatest variance in Y (highest adjusted R^2^ value) were considered the most predictive. Variable Importance in the Projection (VIP) values were used to evaluate the contribution of each variable, with VIP > 0.5 considered significant [35] and VIP > 1 indicating the most relevant predictors of the outcome [34,36].

## 3. Results

### 3.1. Sociodemographic, Burn Injury, and Clinical Parameters of Patients with Mild and Severe Inhalation Injury

**Figure 2:**
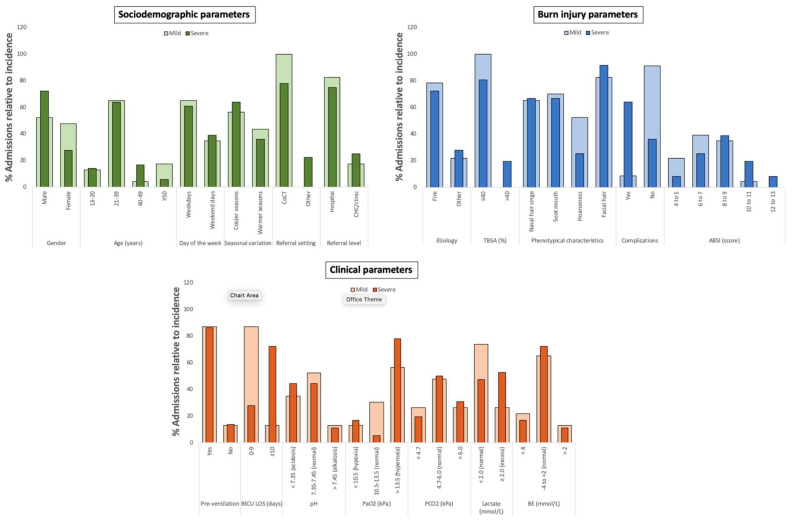
Sociodemographic, burn injury and clinical parameters of patients (*n* = 59) with mild and severe inhalation injury admitted to TBH’s WCPATBC from 23 April 2016 and 15 August 2017 (CoCT—City of Cape Town, CHC—community health center, TBSA—total body surface area, ABSI—Abbreviated Burn Severity Index, BICU LOS—burns intensive care unit length of stay, PaO_2_—arterial oxygen partial pressure, PCO_2_—arterial carbon dioxide partial pressure, BE—base excess).

Of the 62 patient files initially screened, 3 were excluded due to incomplete data, leaving 59 patients for analysis. Patients with severe inhalation injury represented 61% of the total admissions (mean 11.2 total ventilation days; CI = 9.5–12.9), with 14 of the 15 deceased patients presenting with the severe form. The latter presenting with a mortality rate of 38.9% compared to the mild form of 4.3% (Table 1). A 2.6-fold predominance was observed with males and severe injury (*p* = 0.000), while the 21–39-year age group was most affected by both degrees with near identical mean ages (mild, mean = 32.6 years; CI = 27.5–37.6; *p* < 0.032 and severe, mean = 32.9 years; CI = 29.5–36.5; *p* < 0.001). Both degrees were also predominantly sustained on weekdays, during the colder seasons, with flame etiology and in patients referred from hospital settings and within the CoCT district (*p* ≤ 0.043). TBSA ≤ 40% accompanied all mild (mean = 16.3%; CI = 11.5–21.2) and most of the severe cases (mean = 80,6%; CI = 22.5–33.1; *p* = 0.000). Regarding the four phenotypical characteristics, the same median values were observed for injury degrees (mild, median = 3.0; IQR = 2.0–4.0, and severe, median = 3.0; IQR = 2.0–3.0). Facial burns were most frequently observed (mild: 82.6%; severe: 91.7%), and hoarseness the least (mild: 52.2%; severe: 25.0%). Severe inhalation injury-related patients presented with the most complications (>63%; *p* = 0.000) while septic shock was observed in only two patients with mild injury (Figure 2). VAP was the second most common complication, present in seven patients, while concurrent ARDS and septic shock occurred in nine. Patients in the severe injury group also had more ABSI scores in serious to maximum ranges (median = 8.3 ABSI score; IQR: 7.6–8.9).

Both injury groups had a high and similar (*p* = 1.000) occurrence of ventilation prior to admission (mild: 87%; *p* = 0.002 and severe: 86.1%; *p* = 0.000). Patients with mild inhalation injury (87.0%) had shorter BICU LOS (≤9 days, median = 5.0; IQR = 3.0–6.0; *p* = 0.001) while severely affected patients spent a min of 10 days (mean = 12.0 days; IQR = 9.0–11.0; *p* = 0.000) in the BICU. With the exception of elevated PaO_2_ levels (56.5%; mean = 16.8 kPa; CI = 14.1–19.6; *p* = 0.000), majority of the patients with mild inhalation injury had normal ranges for the remaining ABG parameters, these included: pH (52.2%; mean = 7.4; CI = 7.3–7.4), PCO_2_ (47.8%; mean = 5.3 kPa; CI = 4.8–5.9), Sats (87.0%; median = 98.0%; IQR = 96.0–99.0), lactate (73.9%, median = 1.4 mmol/L; IQR = 0.7–2.4), and BE (65.2%; mean = −2.3 mmol/L; CI = −3.8 to −0.7) (*p* ≤ 0.032). With the severe inhalation injury cases, the following blood gas parameters were within the normal ranges: PCO_2_ (50.0%; mean = 5.5 kPa; CI = 5.1–5.9), Sats (88.9%; median = 99.0%; CI = 98.0–100.0), and BE (72.2%; median = −1.7 mmol/L; IQR = −3.7 to 0.1) (*p* ≤ 0.08), whereas elevated PaO_2_ and lactate levels were observed (77.8%; mean = 19.9 kPa; CI = 17.0–22.6; *p* = 0.044 and 52.8%; median = 2.0 mmol/L; IQR = 1.3–2.6; *p* = 0.001, respectively) (Figure 2).

### 3.2. Marked Associations with Sociodemographic, Burn Injury, and Clinical Parameters for Inhalation Injury Presence and/or Degree

Significant associations between inhalation injury and the following factors were observed: %TBSA (*p* = 0.008), complications (*p* < 0.001), and BICU LOS (*p* < 0.001). These variables also demonstrated significant correlation along with ABSI scores, lactate levels, and hoarseness. All correlations were positive, with the exception of hoarseness. BICU LOS and complications had a very strong (rho = 0.908; *p* < 0.001; CI = 0.847–0.945) correlation and a strong correlation (rho = 0.690; *p* < 0.001, CI = 0.522–0.807) with inhalation injury, respectively. The remaining variables were moderately correlated as follows and in descending order: %TBSA (rho = 0.357; *p* = 0.006; CI = 0.103–0.567), ABSI scores (rho = 0.347; *p* = 0.007; CI = 0.093–0.560), lactate levels (rho = 0.331; *p* = 0.011; CI = 0.074–0.556), and hoarseness (rho = −0.314; *p* = 0.015; CI = 0.093–0.560) (Table 2).

### 3.3. Predictive Contribution with Sociodemographic, Burn Injury, and Clinical Parameters for Inhalation Injury Presence and/or Degree

The significantly associated and correlated variables were assessed for their predictive contribution to inhalation injury presence and degree. Hoarseness was excluded from the regression model due to its spurious correlation. ABSI scores were also omitted from the model in order to reduce redundancy, since inhalation injury is included as a parameter in the scoring calculation and would therefore potentially result in biased outcomes. Three regression models analyzed the independent correlated variables, inhalation injury, and their sub-groups. The first model assessed the ungrouped variables for the prediction of inhalation injury presence (Table 3). The second and third models incorporated the sub-groups of these variables as potential predictors for injury presence and severe inhalation injury degree, respectively (Table 4). Severe inhalation injury degree was selected as an additional dependent condition because of its identified close association with more adverse outcomes and mortality.

For all three PLS models, the VIP scores of latent factor 1 were considered based on the most variation in the Y-variables as described by the X-variables, along with the highest R^2^ values. The strongest predictors for inhalation injury presence (R^2^ = 72.5%) were BICU LOS (VIP = 1.495) and complications (VIP = 1.117), while VIP scores of > 0.5 were observed for lactate and %TBSA (Table 3). The regression models incorporating the sub-grouped variables for the prediction of inhalation injury presence (R^2^ = 59.7%) showed that BICU LOS ≥ 10 days (VIP = 1.372) and the presence of complications (VIP = 1.229) were the optimal predictors. It also indicated that TBSA > 40% (VIP = 0.590) and excess lactate levels (VIP = 0.509) were significant contributors (Table 4). These predictive outcomes were similar to those observed for the severe degree (R^2^ = 39%) of inhalation injury. The presence of BICU LOS ≥ 10 days and complications continued to have the highest VIP values (1.303 and 1.229, respectively). TBSA > 40% (VIP = 0.662) and excess lactate levels (VIP = 0.594) remained significant contributors as well (Table 4).

## 4. Discussion

Given the established role of inhalation injury as a co-factor in mortality [7,8], early and accurate identification may help reduce this risk. Despite providing quality care through established protocols and adherence to treatment principles [37,38], resource-limited settings such as the WCPATBC at TBH often face restricted access to diagnostic tools. This highlights the clinical value of utilizing routinely collected medical data for guiding decision-making and prognostication. If supported by statistically significant associations, such data could offer clinicians an accessible, time-efficient tool—particularly if integrated into an electronic framework—before resorting to more invasive diagnostics. Findings from this study demonstrate the potential of patient- and injury-related parameters recorded in medical files to support prognostication of inhalation injury presence or progression. No significant association or correlation was observed with classic symptoms (i.e., singed nasal hairs, perioral soot, or facial burns), despite their inclusion in the standard clinical index of suspicion. This aligned with another study; however, associations with facial burns were observed [39]. A moderate negative correlation with hoarseness was identified, though likely spurious or non-causal—a statistical phenomenon described by Atkinson et al. (2004) and Pearson (1897) [40,41]. This counterintuitive result, where the absence of hoarseness correlated with more severe injury, may reflect limited sample size or unmeasured confounders [42] and thereby contributing to the lack of a notable association. Symptom variability and inconsistent incidence may explain why no single classic symptom reliably predicts injury severity, particularly for more severe forms. While classic signs may suggest smoke exposure, they do not consistently indicate severity. Huang et al. (2022) reported that even in the presence of facial burns, signs like singed nasal hairs and hoarseness lacked predictive value for inhalation injury [43]. However, the authors reported large TBSA as an independent risk factor [43] and a predictor of lower respiratory tract injury [44]. TBSA is also significantly associated with inhalation injury in this study; however, it was not the strongest predictor. Though useful in certain contexts—particularly when inhalation injury is suspected or when considered with factors like etiology and age extremes—TBSA alone is not a definitive marker. Limited mobility, especially in the very young or elderly, may prolong smoke exposure and subsequently increase both burn size and inhalation injury severity. However, smoke exposure without major cutaneous injury remains possible. This mutual exclusivity may explain why TBSA, while predictive, was not the strongest independent marker in this study.

BICU LOS, complications, and elevated lactate levels were additional parameters significantly associated with inhalation injury. Prolonged BICU LOS and the presence of complications were the strongest predictors in this cohort; these associations aligned with previous findings [14,45,46,47,48]. The most frequent complications were VAP and ARDS with septic shock, particularly among patients with severe inhalation injury. Monteiro et al. (2017) identified complications such as ARDS and pneumonia as independent predictors of mortality in patients with inhalation injury, with ARDS having the greatest prognostic impact [44]. ARDS reportedly occurred in a notable proportion of burn patients [49], but it is more frequent in those with inhalation injury [50]. Together with pneumonia, ARDS contributes significantly to the respiratory dysfunction associated with inhalation injury [51,52], and, when induced by it, tends to cause more severe oxygenation deficits than ARDS from cutaneous burns alone [53]. Septic shock, the final and most severe stage of sepsis, was another prevalent complication, consistent with studies linking inhalation injury to increased sepsis risk due to acute lung injury [6,54,55]. VAP, frequently observed in lower airway injuries [44], was another prominent complication in this cohort, reinforcing the severity of inhalation injury. The frequent occurrence of pneumonia further supports this observation. Whether one complication precipitates another remains unclear and warrants investigation into potential compounding effects as part of the injury’s sequelae. However, because complication development depends on immunological responses and injury progression [56,57], they often do not appear immediately after injury or soon after admission. Thus, complications are unlikely to serve as early prognostic markers unless present at admission—a scenario more common in delayed referrals or prolonged transport. Nonetheless, their presence may indicate both the likelihood and severity of inhalation injury, particularly in cases of initial underestimation or misdiagnosis. These findings suggest that referral delays and complication onset may serve as indirect indicators of injury progression and could be considered in future prognostic frameworks. Complications also occurred more frequently in patients who received mechanical ventilation [58], who in this study represented individuals with more severe inhalation injuries. Mechanical ventilation is typically employed to treat oxygen-related abnormalities [59], including perfusion deficits and metabolic disturbances such as lactic acidosis [60]. Among the 36 patients at the WCPATBC with severe inhalation injury, approximately 53% presented with hyperlactatemia (lactate ≥2 mmol/L), and five developed lactic acidosis (≥4 mmol/L). Elevated lactate levels (>2 mmol/L) have been consistently linked to poor burn outcomes [61,62], but are more often proposed as mortality markers [61,62,63,64] rather than indicators of inhalation injury. Although not typically studied in this context, elevated lactate may relate to inhalation injury through mechanisms such as cellular hypoxia [65] and cyanide toxicity [66], both common sequelae of smoke inhalation. Hyperlactatemia has also been associated with septic conditions [67,68], offering a plausible explanation for the high incidence of septic shock among patients with severe inhalation injuries in this cohort.

The interrelated nature of clinical markers in inhalation injury was a key observation in this study, as one factor often exacerbates or perpetuates another. Consequently, no single marker can serve as a definitive prognosticator unless present immediately post-incident, on admission, or shortly thereafter. An important focus of this study is identifying markers available at or shortly after admission to guide early clinical decision-making. Lactate levels, measurable soon after presentation, provide such immediate prognostic value. In contrast, BICU length of stay and the development of complications, while strongly associated with inhalation injury severity, are inherently time-dependent and therefore reflect disease progression rather than early prognostic potential. Although these time-dependent parameters cannot serve as early prognostic markers, their inclusion in the analysis is justified for several reasons: (1) they are consistently reported in the literature as indicators of inhalation injury presence and severity; (2) they are routinely documented in medical files, making them readily available for reliable retrospective analysis; and (3) their strong associations in our cohort highlight outcome burden while providing a comparative baseline for evaluating the potential value of earlier prognostic markers. Taken together, these considerations highlight the importance of distinguishing between markers available at admission and those that manifest later, ensuring their appropriate interpretation in clinical practice. Distinguishing between early-available markers and time-dependent indicators is crucial for applying these findings in clinical practice, particularly in resource-limited environments where rapid risk stratification is essential. TBSA may offer prognostic value when interpreted alongside relevant contextual factors such as exposure duration, burn etiology, and mobility-related comorbidities—several of which may be absent from initial clinical documentation. Among the early-available markers, lactate warrants particular attention, as understanding its underlying physiological mechanisms is critical for interpreting its prognostic significance in inhalation injury. Elevated lactate may result from both anaerobic and aerobic processes: the former associated with hypoxia and sepsis [68,69], and the latter representing a physiological response to increased metabolic demand [70,71]. Differentiating these pathways is essential when considering lactate as a marker for inhalation injury. Importantly, dynamic lactate trends provide greater prognostic insight than isolated measurements [72,73]; serial monitoring could help delineate changes from injury onset through to outcome, supporting the development of time-based cut-off concentrations for prognostic use. Lactate clearance also warrants consideration since efficient clearance within 24 h has been associated with improved survival [74], while failure to clear excess lactate allows hyperlactatemia to persist [69]. As these mechanisms aim to restore homeostasis, understanding the clearance timeline may further enhance the clinical interpretation of lactate levels in relation to inhalation injury severity. While these findings offer valuable insight, several limitations should be acknowledged. The study’s retrospective design and single-center setting, combined with a relatively small sample size, may limit generalizability. However, this cohort reflects the total number of eligible admissions during the approved study period at the country’s only adult specialist burn center, where patient volume will be inherently constrained by strict admission criteria and variable LOS based on injury severity. Despite the modest sample size, the strong associations observed highlight the robustness of the analyses and offer a benchmark for future studies in similar populations or resource-constrained settings. It should also be noted that although the data are from 2016 to 2017, this historical dataset remains scientifically valuable: it provides a reference point for comparative analysis over time, allows trends and shifts in disease and injury management to be assessed, and enables alignment with the existing literature to reinforce reliability. Incomplete medical files were excluded to preserve statistical integrity, which restricted some analyses but ensured methodological robustness. While data completeness was largely adequate for descriptive reporting, analyses requiring pairwise comparisons—such as correlation and regression—were affected by missing values. Moreover, manual extraction by clinical personnel, while ethically appropriate and compliant with governance protocols, may have introduced potential for human error or omissions. These challenges highlight the need for a standardized and streamlined clinical data capture system tailored to high-acuity environments. Embedding research-aligned documentation within routine clinical workflows may improve both data quality and research utility without overburdening staff. Additionally, sampling occurred at a single time point on or shortly after admission. While this supports early prognostication, serial monitoring could have better delineated temporal dynamics and clarified whether observed changes were directly injury-induced or part of compensatory physiological responses. Future studies incorporating longitudinal data and larger, multi-center cohorts may help validate these findings, refine the prognostic value of early clinical markers, and identify additional correlated parameters that may have been previously overlooked.

## 5. Conclusions

Burn survivability models often rely on early indicators from patient history and physical examination, many of which also inform the clinical diagnosis of inhalation injury. Given this overlap, routinely collected parameters may hold prognostic value for identifying inhalation injury—an established contributor to burn-related mortality. This study identified four variables with significant associations: BICU LOS, complications, %TBSA, and blood lactate concentration. BICU LOS and complications were the strongest predictors, though both are time-dependent and may not reflect early severity. While %TBSA was also associated with inhalation injury, it may lack standalone prognostic strength. Lactate levels, measurable shortly after admission, fulfilled the criteria for early prognostication most robustly, but should be assessed dynamically and in relation to clearance rates. Importantly, the study affirms that no single clinical parameter provides absolute prognostic certainty, but the combination of factors may reflect evolving injury severity. Recognizing these patterns may support earlier risk stratification and guide clinical decisions for targeted interventions, especially in resource-limited settings. Future prospective studies involving larger, more diverse cohorts are needed to validate these findings, refine cut-off thresholds, and assess the clinical utility of integrating such markers into electronic triage and prognostic tools to enhance early detection and treatment planning.

## Figures and Tables

**Table 1:** Mild and severe inhalation injury-related admissions and mortality.

	Mild	Severe
Central indices (Mean, 95% CI)	2.7 (2.3–3.4)	11.2 (9.5–12.9)
Total admissions (*n* = 59)	23	36
% mortality relative to admissions	4.3	38.9
Mortality (*n* = 15)	1	14
% mortality relative to mortality incidences	6.7	93.3

**Table 2:** Association and correlation between study variables and inhalation injury.

Study Variables	Fisher’s *p*-Value	Correlation Coefficient (rho)	rho *p*-Value	95% CI
**Sociodemographic factors**				
Gender (male/female)	0.350	−0.049	0.712	−0.308 to 0.217
Age (years)	0.080	0.095	0.474	−0.173 to 0.350
Day of the week (week/weekend days)	0.379	−0.006	0.963	−0.269 to 0.258
Seasonal variation (colder/warmer)	0.421	−0.013	0.920	−0.276 to 0.251
Referral setting (CoCT/other districts)	0.161	0.151	0.252	−0.117 to 0.399
Referral Level (hospitals or CHC/clinics)	0.082	0.107	0.419	−0.161 to 0.360
Days between injury & admission (days)	0.311	−0.109	0.409	−0.289 to 0.238
**Injury characteristics**				
Etiology (fire/other sources)	0.351	0.118	0.373	−0.150 to 0.370
TBSA (%)	0.008*	0.357	0.006 **	0.103–0.567
Singed nasal hairs (yes/no)	0.472	0.068	0.611	−0.199 to 0.325
Soot around/in mouth (yes/no)	0.908	0.046	0.730	−0.220 to 0.306
Hoarseness (yes/no)	0.178	−0.314	0.015*	−0.533 to 0.055
Facial burns (yes/no)	0.750	0.091	0.492	−0.176 to 0.346
Complications (yes/no)	<0.001 *	0.690	<0.000 **	0.522–0.807
ABSI scores (4−13 points range)	0.163	0.347	0.007 **	0.093–0.560
**Clinical parameters**				
BICU LOS (days)	<0.001 *	0.908	<0.001 **	0.847–0.945
Ventilation prior to admission (yes/no)	0.267	0.019	0.887	−0.246 to 0.281
pH	0.936	−0.223	0.090	−0.399 to 0.116
PaO_2_ (kPa)	0.381	0.006	0.964	−0.258 to 0.269
PCO_2_ (kPa)	0.955	−0.089	0.503	−0.179 to 0.344
Sats (%)	0.626	0.200	0.129	−0.067 to 0.440
Lactate (mmol/L)	0.060	0.331	0.011 *	0.074 to 0.556
Base excess (mmol/L)	0.936	−0.040	0.763	−0.300 to 0.226

CI—confidence interval, rho—Spearman’s Rank coefficient, CoCT—City of Cape Town, CHC—community health center, TBSA—total body surface area, ABSI—abbreviated burn severity index, BICU LOS—burns intensive care unit length of stay, PaO_2_—arterial oxygen partial pressure, PCO_2_—arterial carbon dioxide partial pressure. Interpretation of rho [31] −0.01 to 0.19 (no or negligible); 0.20 to 0.29 (weak); 0.30 to 0.39 (moderate); 0.40 to 0.69 (strong); ≥0.70 (very strong). *: Correlation is significant at the 0.05 level (2-tailed). **: Correlation is significant at the 0.01 level (2-tailed).

**Table 3:** Predicted variances and VIP values incorporating the significantly associated and correlated independent variables for the inhalation injury presence.

**Predicted variances of latent factors for inhalation injury presence**				
	**1**	**2**	**3**	**4**
*X*-variance	0.509	0.216	0.087	0.188
*Y*-variance	0.729	0.082	0.008	0.000
Adjusted R-square (R^2^)	0.725	0.805	0.809	0.806
**VIP values per variable for inhalation injury presence prediction**				
Complications	1.117	1.064	1.070	1.070
%TBSA	0.487	0.606	0.609	0.609
BICU LOS	1.495	1.491	1.487	1.487
Lactate	0.527	0.544	0.542	0.543

VIP—variable importance in prediction, TBSA—total body surface area, BICU LOS—burns intensive care unit length of stay.

**Table 4:** Predicted variances and VIP values incorporating the sub-groups of the significantly associated and correlated independent variables for the inhalation injury presence and severity.

**Predicted variances of latent factor 1 for inhalation injury presence and severe degree**		
	**Inhalation injury presence**	**Severe inhalation injury**
*X*-variance	0.490	0.492
*Y*-variance	0.604	0.401
Adjusted R-square (R^2^)	0.597	0.390
**VIP values per variable sub-groups for injury presence and severe degree prediction**		
Complications (present)	1.229	1.229
%TBSA (>40 days)	0.590	0.662
BICU LOS (≥10 days)	1.372	1.303
Lactate (Excess)	0.509	0.594

VIP—variable importance in prediction, TBSA—total body surface area, BICU LOS—burns intensive care unit length of stay.

## Data Availability

The data presented in this study are available on request from the corresponding author due to the inclusion of identifiable clinical information recorded in the patient’s medical file, which is subject to confidentiality and ethical restrictions.

## Abbreviations

The following abbreviations are used in this manuscript:

TBSA	Total body surface area
BICU LOS	Burns intensive care unit length of stay
VIP	Variable Importance in the Projection
WCPATBC	Western Cape Provincial Adult Tertiary Burns Centre
TBH	Tygerberg Hospital
WC	Western Cape
CoCT	City of Cape Town
ABSI	Abbreviated Burn Severity Index
BICU LOS	Burn Intensive Care Unit Length of Stay
ABG	Arterial blood gas
BE	Base excess
